# Delivering vaping cessation interventions to adolescents and young adults on Instagram: protocol for a randomized controlled trial

**DOI:** 10.1186/s12889-022-14606-7

**Published:** 2022-12-10

**Authors:** Joanne Chen Lyu, Sarah S. Olson, Danielle E. Ramo, Pamela M. Ling

**Affiliations:** 1 Center for Tobacco Control Research and Education, University of California, San Francisco, San Francisco, CA USA; 2 Division of General Internal Medicine, Department of Medicine, University of California, San Francisco, San Francisco, CA USA; 3 HopeLab, 100 California St #1150, San Francisco, CA 94111 USA

**Keywords:** Adolescents and young adults, Electronic nicotine delivery system (vaping) cessation intervention, Social media, Randomized controlled trial

## Abstract

**Background:**

Adolescent and young adult use of electronic nicotine delivery systems (“vaping”) has increased rapidly since 2018. There is a dearth of evidence-based vaping cessation interventions for this vulnerable population. Social media use is common among young people, and smoking cessation groups on social media have shown efficacy in the past. The objective of this study is to describe the protocol for a randomized controlled trial (RCT) testing the efficacy of an Instagram-based vaping cessation intervention for adolescents and young adults.

**Methods:**

Adolescents and young adults aged 13–21 residing in California who have vaped at least once per week in the past 30 days will be recruited through social media ads, community partners, and youth serving organizations. Participants will be randomly assigned to intervention or control conditions: the intervention group takes place on Instagram, where participants receive up to 3 posts per weekday for 25 days over 5 weeks; the control group will be directed to kickitca.org, a website offering links to chatline and texting cessation services operated by the California Smokers' Helpline. The primary outcome is biochemically verified 7-day point prevalence abstinence for nicotine vaping; secondary outcomes are vaping reduction by 50% or more, vaping quit attempts, readiness to quit vaping, confidence in ability to quit, desire to quit, commitment to abstinence, and use of evidence-based cessation strategies. Both the primary outcome and secondary outcomes will be assessed immediately, 3 months, and 6 months after the treatment.

**Discussion:**

This is the first RCT to test a vaping cessation intervention delivered through Instagram. If effective, it will be one of the first evidence-based interventions to address vaping among adolescents and young adults and add to the evidence base for social media interventions for this population.

**Trial registration:**

ClinicalTrials.gov: NCT04707911, registered on January 13, 2021.

**Supplementary Information:**

The online version contains supplementary material available at 10.1186/s12889-022-14606-7.

## Background

The aggressive promotion and uptake of electronic nicotine delivery systems (colloquially called “vaping”) among youth in the US [1–9] have led to an increase in overall nicotine use [8, 10–13]. Among high school students, vaping increased 135% from 11.7% to 27.5% between 2017–2019 [8, 13]. Despite a documented decline in e-cigarette use among young people during the COVID-19 pandemic [14], in 2021, e-cigarettes continued to be the most commonly currently used tobacco product with 11.3% of high school students and 2.8% of middle school students reporting use in the past 30 days[15]. Vaping among young people is especially concerning because compared with older adults, the brains of adolescents and young adults are more vulnerable to the harmful health effects of nicotine that most e-cigarettes contain [16, 17]. In addition, e-cigarettes introduced in recent years have the potential to deliver equal or more nicotine compared to tobacco cigarettes [18], potentially increasing their addictiveness. Prevention programs to address adolescent vaping include prevention media campaigns and educational classroom materials, such as the Stanford Tobacco Prevention Toolkit [19] and the CATCH my breath e-cigarette educational program [20]. Fewer programs address vaping cessation for adolescents and young adults: telephone counseling and texting programs are offered [21, 22], and Truth Initiative offers a free texting vaping intervention that has shown efficacy in a randomized trial [23].

Social media have been a part of life for young Americans with 95% of adolescents and 84% of adults ages 18 to 29 ever using social media [24, 25]. While aggressive targeted marketing of e-cigarettes on social media such as Twitter, Instagram, and YouTube significantly contributes to high awareness of e-cigarettes and high e-cigarette use rates among youth [26, 27], social media are also a promising channel to reach a large number of adolescents and young adults who vape without geographical restrictions to deliver vaping cessation interventions. Social media-based smoking cessation interventions for adults have demonstrated feasibility, acceptability, and early efficacy [28, 29]. However, there are few effective and high-quality smoking cessation interventions for adolescents and young adults [30, 31] and even less evidence on social media interventions to address vaping. To the best of our knowledge, none of the existing vaping cessation interventions for adolescents and young adults are delivered via social media. To fill this gap, we will conduct a randomized controlled trial (RCT) to test the efficacy of a social media-based intervention designed specifically to support cessation among adolescents and young adults who vape. Though vaping cessation interventions are relatively new and rigorously tested programs are limited, a meta-analysis of research on adolescent cigarette smoking cessation interventions showed that cessation programs increased the probability of quitting by approximately 46%, with higher quit rates in programs that included a motivation enhancement component, cognitive-behavioral techniques, and social influence approaches [32]. This suggests that vaping inventions utilizing these strategies may be effective to address the problem of vaping among young populations. One model has incorporated Motivational Interviewing (MI), cognitive behavioral coping skills, and the Transtheoretical Model (TTM) of behavior change (i.e., readiness to quit) to create the Tobacco Status Project (TSP) [31, 33–35], a smoking cessation intervention for young adult smokers implemented entirely through private Facebook groups. Participants in TSP joined private Facebook groups where they received daily posts and were encouraged to interact with each other for 90 days [33]. Biochemically verified 7-day point prevalence abstinence at the 3-month follow-up was significantly higher in the intervention group (8.3%) compared to the control group referred to Smokefree.gov (3.2%) [33]. In our current study, we will deliver intervention materials adapted from TSP to address nicotine vaping, the adolescent and young adult target audience, and to deliver the program via the Instagram platform, which is more popular than Facebook among adolescents, being used by 72% of adolescents in 2018 [36].

## Objectives

This study will evaluate the efficacy of the Instagram-based vaping cessation intervention among adolescents and young adults in an RCT. The primary aim is to test the efficacy of the intervention to achieve biochemically verified nicotine vaping abstinence when the treatment ends and sustain it for 6 months after the treatment. The secondary aim is to test the effectiveness of the intervention in terms of decreasing vaping frequency, and increasing quit attempts, readiness to quit, confidence in ability to quit, desire to quit, commitment to abstinence, and use of evidence-based cessation strategies assessed immediately, 3 months, and 6 months after the treatment.

## Methods/design

### Study design

This is an RCT with an intervention group and a control group. This study protocol was written according to SPIRIT (Standard Protocol Items: Recommendations for Interventional Trials), a guideline that defines standard protocol items for clinical trials and is widely endorsed as an international standard for trial protocols [37, 38]. The completed SPIRIT checklist is shown as Additional file 1.

### Participants

Adolescents and young adults aged 13–21 residing in California who have vaped at least once per week in the past 30 days will be recruited online via Facebook and other social media, augmented by outreach through community partners and youth serving organizations (the detailed recruitment procedure is elaborated in the Recruitment section below). This study focuses on adolescents and young adults aged 13–21 because people in this age group both need vaping cessation support and have integrated social media into daily life. Children under the age of 13 are not included because the intervention takes place on social media and popular sites all set their age limit at 13, in compliance with the Children’s Online Privacy Protection Act [39]. There are no exclusions by race/ethnicity or gender or sexual orientation. Those who are pregnant and/or currently use other tobacco products will not be excluded from participation, as they may benefit from vaping cessation.

### Inclusion criteria

- 1. English literacy;
- 2. Age between 13-21 years;
- 3. Indicate they use social media “most” (≥ 4) days per week;
- 4. Have vaped at least once per week in the past 30 days;
- 5. Access to a computer or mobile phone with photo capability to verify abstinence from vaping;
- 6. Indicate they are considering quitting or are interested in quitting within the next 6 months;
- 7. Currently reside in California. This is because the funder for this study, the California Tobacco Related Diseases Research Program, requires the research be conducted in California.

### Exclusion criteria

- 1. No English literacy;
- 2. Age under 13 or over 21 years;
- 3. Insufficient social media use (3 or fewer days per week);
- 4. Have not vaped at least once per week in past 30 days;
- 5. No access to computer or mobile phone with photo capability to verify abstinence from vaping;
- 6. Not interested in or considering quitting within the next 6 months;
- 7. Not California residents.

### Sample size estimation

Power analyses were generated using the two-group repeated proportions module in NCSS PASS 14 [40] to compute minimum detectable effect sizes for the primary analysis. Assuming α = 0.05, power = 0.80, rho = 0.43 and a 7-day abstinence base rate of 3.2% and a quit rate of 8.3% in the treatment group from our preliminary data [31], group sample size of 234 (N_total_ = 468) is needed. Assuming a dropout rate of 7%, we need 500 participants.

### Recruitment

**Fig. 1:**
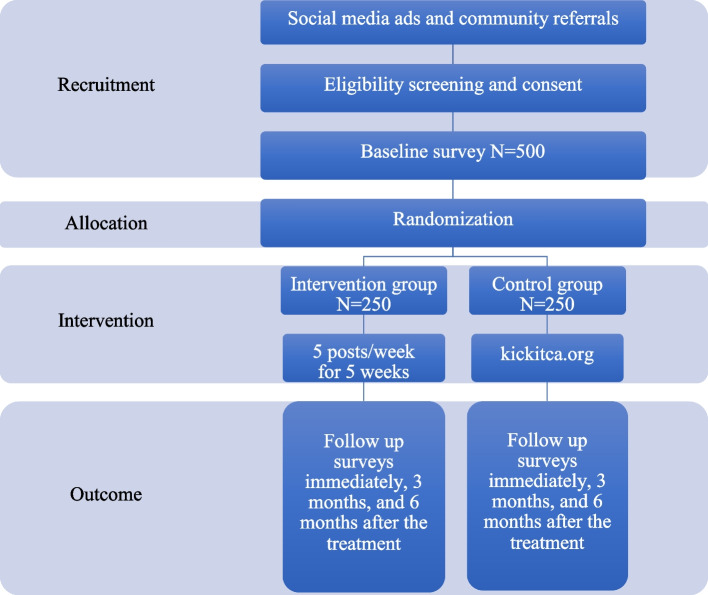
Participant flow through the randomized clinical trial

A total of 500 participants will be recruited using social media campaign strategies that have been successfully employed in previous online survey and intervention studies [31, 41], augmented by outreach through community partners and youth serving organizations. Advertisements will be targeted based on age and with keywords likely to reach e-cigarette and social media users. Ads will include an image and short text consistent with most social media advertising guidelines. Most ads will mention study incentive. Ad spending and response (clicks on ads and study enrollment numbers) will be monitored with adjustments as needed to maximize efficiency of spending. Ads will include a link to the study’s website www.quitthehitca.com, which has a short description of the study and eligibility questions that address the inclusion criteria and exclusion criteria. If respondents are eligible, then they will be taken to the informed consent page. The participant flow through the RCT is shown in Fig. 1.

### Study procedure

The state of California allows teenagers to seek treatment for substance use without parental consent [42]. Parental consent may pose a risk to teens by revealing their vaping behavior, which may make them reluctant to engage in vaping cessation interventions. Therefore, in this study, both young adult participants and adolescent participants will consent to participate in the research themselves. Participants will complete consent electronically on the study website immediately following completion of eligibility screening. The consent forms will contain a thorough description of the research project, including the procedures involved, the benefits and risks, compensation, the right to non-participation, and the Research Subject’s Bill of Rights. These documents are also available to participants at any time on the study website: www.quitthehitca.com. The amount of text in the standard consent form is burdensome to read on a mobile device, and participants may tend to skip the form. To enhance usability, especially for mobile devices, a shortened version of the consent form and a video version of the consent with key elements have been created for mobile device review and consent. Participants on mobile devices will also have the opportunity to download and save the full consent form for future viewing.

Information about the informed consent process is written in clear, concise English that can be understood at a 6th-grade reading level. Participants will be asked to contact study staff if they do not understand any part of the informed consent information.

Before being able to proceed with the study, participants will indicate on an electronic form that they understand what this study is about, that their participation is voluntary, that they are not required to complete any part of this study, and that there are risks associated with study participation. Incomplete or incorrect answers will result in the potential participant being prompted to review the full consent document before attempting the consent questions again. Potential participants will not be enrolled in the study if they fail to complete the form four times. In both the screening and baseline survey, participants are asked about location questions (zip code in the screening and county in the baseline survey). Our research staff will check the consistency of their answers and contact the participants with inconsistent answers using the contact information they provided to confirm the authenticity of the participants. In addition, participants must follow the study account on Instagram, and study personnel will review the profiles of followers for consistency with age and California location. Irregular or inconsistent answers or participants otherwise having difficulty completing the form will be contacted by trained study personnel who will review the consent material and assist with study enrollment. Study personnel may detect behavior, responses or Instagram accounts that do not appear to be authentic. Fraudulent participants identified through this process will be removed and documented. In the consent form, participants will be asked to remain in the study’s Instagram group throughout the duration of the study (about 7 months) for the intervention and for follow-up contacts. Participants will be paid $10 in the form of an electronic gift card for completing the baseline assessment. Participants will be paid $15–35 for each follow-up assessment for a possible total of $100.

Participants who sign the consent form will be asked to create a login, and then redirected to the baseline assessment. This study will recruit 500 participants in total. Those completing the baseline assessment are randomized using a computer-generated random sequence which automatically assigns participants without revealing allocation to study investigators. Participants are assigned to either the Instagram intervention or the control condition, which is a referral to free support administered by the California quitline via the website kickitca.org. Approximately two weeks after the first participant is enrolled in an intervention group, the group will begin to receive the intervention program. Groups may start slightly sooner or later than 2 weeks based on the pace of recruitment, aiming for a group size between 5 – 15 participants. Depending on the pace of enrollment, multiple groups may run simultaneously.

### Development of the intervention

This intervention was adapted from the TSP described above. The TSP is a smoking cessation intervention for young adults that was delivered through private Facebook groups and utilized the US Clinical Practice Guidelines for smoking cessation [43] and the TTM of behavior change [44, 45], with counseling messages tailored to participants’ readiness to quit delivered in 90 Facebook posts over 90 days along with weekly live counseling chat sessions with a trained smoking cessation counselor. The first adaptation of the TSP by our team was to tailor intervention content for sexual and gender minority (SGM) young adult smokers and had preliminary evidence for the effectiveness: an SGM-tailored Facebook smoking cessation intervention increased reported abstinence from smoking, compared to a non-tailored intervention [46]. This study followed the similar adaptation approach as the SGM project to tailor the intervention content for adolescents and young adults who vape: revising the intervention based on extensive iterative formative work. We have an established partnership with HopeLab (a social innovation lab focusing on designing science-based technologies to improve the health of teens and young adults) and with Rescue (a marketing agency providing behavior change marketing services) for intervention adaptation. Before our study, Rescue conducted 18 focus groups and 66 interviews with 269 adolescents to develop adolescent vaping messaging for cessation and interviewed 380 adolescents with vaping experience. Our partners worked intensively in 2021 to further develop the Instagram intervention for adolescents who vape, including co-creation sessions working with 9 adolescents. Surface-level tailoring [47] was accomplished using pictures of adolescents who vape, as well as symbols and terms that were meaningful to the group. Deep-level tailoring [47] involved discussion of adolescent issues relevant to vaping. Examples included increasing awareness of predatory marketing targeting young people, awareness and experiences of addiction, and education about the gateway effect of vaping on smoking initiation. Two design sprints and development of the websites and infrastructure were completed prior to launching a test version of the intervention. 90 adolescents who vape participated in the pilot tests to address the feasibility, acceptability, usability, and helpfulness of the test version. Participant surveys included ratings of different elements of the intervention, and interviews addressed their experience participating in different aspects of the intervention, what they liked most and least about the experience, and identified which aspects of the intervention or procedures to retain or change. The partner team met virtually on a weekly basis throughout the development process until January 2022.

#### Intervention group

The vaping cessation intervention will be implemented on Instagram. Participants in the intervention group will be assigned to groups on Instagram, where they will receive up to 3 posts per weekday for 25 days (5 weekdays, no weekends) over 5 weeks. Posts incorporate skills from cognitive behavioral therapy, found effective for long-term smoking cessation [48], as well as the TTM processes of self-liberation (e.g., making a commitment to quit), stimulus control (e.g., removing vaping paraphernalia from the home), and counter conditioning (e.g., engaging in alternative behaviors). Posts also encourage setting a quit date and making a detailed quit plan. Posts include a combination of images, videos, and text to elicit a response from participants. Posts may suggest that participants use their social media or real social networks for support with vaping cessation. However, they are not required to share any information about substance use on social media.

Groups are facilitated by a trained Guide, a certified cessation counselor with over 4 years of experience facilitating smoking cessation groups on Facebook, working with the Principal Investigator and Sub-Investigators. The Guide will send the daily posts in groups. In addition, the study employs a pediatrician on demand if additional expertise or clinical advice is needed. Participants will be educated about signs of nicotine dependence and if they express interest in pharmacotherapy, we will encourage them to access this through their personal healthcare providers.

#### Control group

Participants in the control condition will be directed to kickitca.org, a website offering links to chatline and texting cessation services operated by the California Smokers' Helpline, the free state quitline program that supports smoking, vaping, and smokeless tobacco cessation strategies.

### Baseline measures

Baseline measures will consist of sociodemographic characteristics, nicotine vaping, quitting experience, motivations to quit (such as readiness to quit vaping, confidence in ability to quit, desire to quit, and commitment to abstinence), other tobacco use, marijuana/cannabis use, social media use integration, and symptoms of depression or anxiety.

### Outcome measures

#### Primary outcome

7-day point prevalence abstinence will be assessed immediately, 3 months, and 6 months after the treatment. Participants reporting no nicotine vaping in the past 7 days will be coded as abstinent. At each follow-up assessment, those reporting 7-day abstinence and not using NRT will be mailed a saliva cotinine test kit for biochemical verification. Participants will be provided both written instruction and a video demonstrating how to correctly perform the saliva cotinine test, and take photos of themselves doing the test and a photo of the result, and how to send the photos to our research staff. Participants with a salivary cotinine level < 20 ng/ml will be considered to have achieved biochemically verified nicotine vaping abstinence.

#### Secondary outcomes

We will measure seven secondary outcomes as below:

• Vaping reduction by 50% or more between baseline and each follow-up will be calculated at each time point by multiplying the number of vaping days in the past 7 days and the number of puffs or hits they took on an average day.
• Vaping quit attempt will be measured by questions asking the presence and the number of quit attempts lasting at least 1 day since the last assessment.
• Readiness to quit vaping will be assessed using the Stages of Change Questionnaire [42] to classify participants as in Contemplation or Preparation phases [45]. Outcome will be measured as proportion change in Stage at assessments conducted immediately, 3 months, and 6 months after the treatment.
• Confidence in ability to quit will be measured by asking how confident the participants are about avoiding vaping nicotine in the next 6 months.
• Desire to quit will be measured by asking how much the participants want to quit vaping.
• Commitment to abstinence will be assessed with the Thoughts About Abstinence Form [49], categorizing their goal as no goal, intermediary goal (e.g., reduced vaping), or total abstinence. Outcome will be measured as proportion endorsing a goal of abstinence at each time point.
• Use of evidence-based cessation strategies will be measured by asking the supports participants used when they tried to quit in the past (30 days/3 months).

### Data collection and management

The three follow-up assessments conducted immediately, 3 months, and 6 months after the treatment will be administered online using Qualtrics through UCSF MyAccess, which includes Secure Sockets Layer encryption, and adheres to HIPAA standards, allowing encrypted transmission of all survey data. Research staff will send emails, text messages, and messages in Instagram with the survey links to notify participants of assessments. At most three emails will be sent to remind participants of the assessments. Those who don't respond after the three contact attempts are counted as dropout at each time point. Those who don't respond in the previous assessments will still be contacted for later assessments. For instance, a participant who did not do the assessment at 3 months post-treatment will still be notified of the assessment at 6 months post-treatment up to three times. Survey data will be securely transferred to password-protected Excel or SPSS files that will be securely stored online using the UCSF Research Analysis Environment, a secure, HIPAA-compliant desktop environment. Files with identifying information will be stored separately from files with other forms of data. Data will only be accessed by the PI and a research assistant until it is de-identified. Study investigators have completed required UCSF training and certification in the conduct of research with human subjects. All collected data will be analyzed and reported in aggregate form for publications. All survey data collection and storage procedures are consistent with current HIPAA guidelines.

In order to ensure and maintain the scientific integrity of this human subject research project, and to protect the safety of its research participants, we will assemble a Data Safety Monitoring Board (DSMB). The DSMB will meet every six months via video conferencing to review the study protocol and procedures. The DSMB will have the responsibility of assuring that participants are not being exposed to unnecessary or unreasonable risks as a result of the pursuit of the study’s scientific objectives.

### Data analysis

Descriptive statistics will summarize sample characteristics and intervention delivery in each group. We will examine treatment condition and baseline descriptive characteristics as predictors of attrition at intervention end and will control for predictors of attrition as covariates in model testing. Missing data will be minimized through online assessment, and subjects will be re-contacted through social media or email to obtain missing information. Two sets of outcome analyses will be conducted: one self-reported by all participants who are maintained in the study, and another based on biochemically verified abstinence rates.

#### Primary analysis

We will use a generalized linear mixed model (GLMM) using the time points immediately, 3 months, and 6 months after the treatment. The model will account for dependence of responses within individuals attributable to repeated measures. GLMMs will include random effects person ID and will be fitted using SAS PROC GLIMMIX with maximum likelihood estimation via adaptive quadrature with a minimum of 15 integration points. Nicotine vaping abstinence will be measured by 7-day point prevalence. Covariates will be sex as a biological variable and other variables that are found to be related to abstinence. If missing data is not negligible, we will use multiple imputation procedures to impute missing data. Data about biochemically verified abstinence will be analyzed using the same method as self-reported 7-day point prevalence of abstinence.

#### Secondary analysis

We will estimate and test mixed effects logistic and multinomial regression models for longitudinal ordinal response data to model secondary outcomes for vaping over time: 1) vaping reduction by 50% or more; 2) vaping quit attempts; 3) readiness to quit vaping; 4) confidence in ability to quit; 5) desire to quit; 6) commitment to abstinence (i.e. endorsing the personal goal of wanting to quit nicotine completely); and 7) use of evidence-based cessation strategies.

### Ethics approval

The study protocol was submitted for ethics approval to WCG IRB, a trusted partner to more than 3,300 research institutions ranging from small community hospitals and research sites to large academic medical centers and universities in the United States. WCG IRB approved this study on August 27, 2021. The protocol is registered with ClinicalTrials.gov (protocol #NCT04707911).

### Research dissemination

Findings of this study will be disseminated to educators and researchers through publications in peer-reviewed journals, presentations at national or international conferences, and sharing in seminars. In addition, we will share results with community partners for this trial including but not limited to reports and meetings with the California Department of Education Tobacco-Use Prevention Education Program and associated programs, the Stanford Tobacco Prevention Toolkit, and partners at the San Francisco Department of Public Health Tobacco Free Project and the California Tobacco Control Program.

## Discussion

This is the first clinical trial of a social media-based vaping cessation intervention for adolescents and young adults. As vaping among young people has become an urgent problem in tobacco control, our study is well timed to provide a rigorously evaluated evidence-based social media intervention to address the epidemic of adolescent and young adult vaping. Using social media platforms to implement the vaping cessation intervention for adolescents and young adults is pioneering. Given the nature of social media, including high use rates among young people and lack of geographic restrictions, the intervention has high potential to reach a large number of young people who vape, provide accessible support, and achieve large scalability. However, though a systematic review of social media interventions for smoking cessation observed greater abstinence, reduction in relapse, and increase in quit attempts [29], social media interventions reported high dropout rates and low engagement over time, which significantly reduced the intervention efficacy [28, 29]. To address this problem, the intervention has been developed with experts in human centered design and co-created by adolescents to maximize acceptability and engagement. We will also utilize state of the art strategies from prior longitudinal studies of adolescents and young adults to track study participants [50, 51].

While social media may be an ideal platform for behavior change interventions targeting adolescents and young adults, the open nature of the social media may comprise a potential threat to individual privacy and unintended information sharing. To protect the participant privacy to the fullest extent possible, the Principal Investigator (Pamela Ling) has obtained a Federal Certificate of Confidentiality to protect the data from subpoena, will make sure that the identifying information (e.g., IP address, email address) will be separated from survey responses, and will give assurances that participants’ e-mail addresses will not be sold or disseminated to any company or individual (participants have been informed of this in the consent form). In addition, group sharing and membership can be limited through privacy settings and individual users can choose who has access to their information. However, the privacy settings and security of material posted on Instagram is under control of Instagram, and thus subject to change should Instagram decide to do so. Therefore, the study investigators cannot guarantee complete privacy of material posted on Instagram. Moreover, at some point during the intervention a participant may share personal information (e.g., vaping quit date, ask for support with reduction or cessation from friends) with their larger social networks. When participants enter the study, they will be informed that the intervention may ask that they share information on social media, but they can choose whether to do so and which people see this information. If participants choose not to share information publicly on social media, it will be made explicit that there will be no consequences whatsoever. In our prior work with Facebook smoking cessation groups, we have not had any privacy concerns among our participants. Any concerns about privacy will be immediately addressed with participants privately.

This intervention is designed for adolescents and young adults, populations in the midst of a stage in life in which they are more susceptible to peer influence [52–54]. Previous studies found that 25% of teenagers aged 16–17 years old posted references to alcohol on their social media profiles [55]; in addition, substance use, violence, sexual behavior, and even suicidality are also commonly displayed on social media platforms [55–58]. This calls for more serious monitoring of daily interaction among the participants in our Instagram groups. First, before starting the groups, we will remind the participants that the focus of the group is on vaping experience and quit vaping efforts, and that interactions should be respectful. Second, we have a trained Guide in the groups, who will monitor the everyday conversation of the participants. If problematic content is spotted, the guide will remind the involved participants privately and report serious cases to the Principal Investigator for resolution and further actions, including study disqualification. One of our inclusion criteria is using social media ≥ 4 days per week. Previous studies have found that depression may be common among adolescents who spend a significant amount of time on online social networks [59]. Another study found that more than half of secondary school students experienced a need for mental health support [60]. Our team is ready to deal with mental health issues and/or with serious withdrawal symptoms such as sleep problems, irritability, or in the unlikely event that they become emotionally distressed as a result of answering assessment questions. The Principal Investigator, a Sub-Investigator, and the Study Physician in this team are all practicing clinicians with experience working with vulnerable populations, young people, and substance use. They can counsel study participants privately over email, or on the phone if necessary and make appropriate referrals to medical providers or mental health resources should participants reveal a need for such support.

This study is the first to implement a vaping cessation intervention for adolescents and young adults entirely through a social media platform and rigorously evaluate it. It is based on a previously tested social media-based intervention for young adult smokers and was developed with thoughtful, iterative, and participant-engaged adaptation. If effective, this study will provide one of the very first evidence-based social media interventions to address the urgent issue of vaping among adolescents and young adults. The program has already been adopted by health departments and other youth serving partners in South Carolina, Minnesota, the San Diego school districts, and Oklahoma, demonstrating it is feasible to deliver this accessible evidence-based support to a large number of adolescents and young adults interested in quitting vaping.

## Supplementary Information

**Additional file 1.** SPIRIT 2013 Checklist: Recommended items to address in a clinical trial protocol and related documents.*

## Data Availability

The datasets generated from the study will be available from the corresponding author upon reasonable request.
